# Early transplantation of human immature dental pulp stem cells from baby teeth to golden retriever muscular dystrophy (GRMD) dogs: Local or systemic?

**DOI:** 10.1186/1479-5876-6-35

**Published:** 2008-07-03

**Authors:** Irina Kerkis, Carlos E Ambrosio, Alexandre Kerkis, Daniele S Martins, Eder Zucconi, Simone AS Fonseca, Rosa M Cabral, Carlos MC Maranduba, Thais P Gaiad, Adriana C Morini, Natassia M Vieira, Marina P Brolio, Osvaldo A Sant'Anna, Maria A Miglino, Mayana Zatz

**Affiliations:** 1 Laboratório de Genética e Imunoquímica, Instituto Butantan, São Paulo, SP, Brasil; 2 Departamento de Cirurgia da Faculdade de Medicina Veterinária da Universidade de São Paulo, SP, Brasil; 3 Genética Aplicada, Atividades Veterinárias LTD, São Paulo, SP, Brazil; 4 Centro de Estudos do Genoma Humano, Departamento de Genética e Biologia Evolutiva, Universidade de São Paulo, SP, Brasil

## Abstract

**Background:**

The golden retriever muscular dystrophy (GRMD) dogs represent the best available animal model for therapeutic trials aiming at the future treatment of human Duchenne muscular dystrophy (DMD). We have obtained a rare litter of six GRMD dogs (3 males and 3 females) born from an affected male and a carrier female which were submitted to a therapeutic trial with adult human stem cells to investigate their capacity to engraft into dogs muscles by local as compared to systemic injection without any immunosuppression.

**Methods:**

Human Immature Dental Pulp Stem Cells (hIDPSC) were transplanted into 4 littermate dogs aged 28 to 40 days by either arterial or muscular injections. Two non-injected dogs were kept as controls. Clinical translation effects were analyzed since immune reactions by blood exams and physical scores capacity of each dog. Samples from biopsies were checked by immunohistochemistry (dystrophin markers) and FISH for human probes.

**Results and Discussion:**

We analyzed the cells' ability in respect to migrate, engraftment, and myogenic potential, and the expression of human dystrophin in affected muscles. Additionally, the efficiency of single and consecutive early transplantation was compared. Chimeric muscle fibers were detected by immunofluorescence and fluorescent *in situ *hybridisation (FISH) using human antibodies and X and Y DNA probes. No signs of immune rejection were observed and these results suggested that hIDPSC cell transplantation may be done without immunosuppression. We showed that hIDPSC presented significant engraftment in GRMD dog muscles, although human dystrophin expression was modest and limited to several muscle fibers. Better clinical condition was also observed in the dog, which received monthly arterial injections and is still clinically stable at 25 months of age.

**Conclusion:**

Our data suggested that systemic multiple deliveries seemed more effective than local injections. These findings open important avenues for further researches.

## Background

Duchenne Muscular Dystrophy (DMD) is a lethal X-linked disorder that affects 1 in 3,500 newborn human males. It is caused by mutations in a large gene located at Xp21 that encodes the muscle protein dystrophin [1]. One third of DMD cases result from new mutations, whereas the remaining are maternally inherited. Affected boys are confined to a wheelchair around age 10 to 12 and later develop respiratory and cardiac problems that lead to death usually in the third or fourth decade.

Satellite cells are spare stem-cells responsible for muscle growth and regeneration. Replacement of defective muscle cells by the patient's satellite cells has been pursued for a long time [2,3]. Other alternative sources of stem cells (SC) such as bone marrow, cord-blood or adipose tissue have been tested in the mdx mouse, which is the murine model for human DMD. These experiments resulted in the incorporation of donor-derived nuclei into muscle, and the partial restoration of dystrophin expression in the affected muscle [4]. However, the mdx mouse does not present evident muscle weakness, with the exception of significant histopathological alterations in the diaphragm [5]. Myoblasts transplantation has also been tried for skeletal muscle tissue engineering [6] but it failed due to the immunogenic properties of these cells. A new strategy to overcome this obstacle has been recently developed through myoblast transplantation tolerance with anti-CD45RB, anti-CD154 and mixed chimerism [7].

The closest model for human DMD is the golden retriever muscular dystrophy (GRMD) dog, which has a splice acceptor site mutation in intron 6, causing a frameshift due to deletion of exon 7 from the mature mRNA [8]. This mutation results in the absence of the muscle protein dystrophin [9].

In the first reported trial using GRMD dogs, bone marrow hematopoietic SC were transplanted from normal litter mates to immunosuppressed GRMD dogs. Nevertheless, dystrophin expression was not restored [10]. In another study, it was suggested that mesoangioblast multipotent cells from dorsal aorta improved muscle function when transplanted into dystrophic dogs [11]. The latter authors used both heterologous wild-type (WT) and autologous genetically modified canine mesoangioblasts in their experiments. In their study, the authors suggested that mesoangioblasts are promising cells to be used in SC therapy for DMD patients. However, because these studies were performed under different regimes of immunosuppression, one cannot rule out that immunosuppression alone may be responsible for clinical improvement as in DMD boys, as it has been suggested in other studies [12].

We recently reported the successful isolation of a population of hIDPSC from dental pulp of non-exfoliated deciduous teeth. Under standard culture conditions, these cells express both the embryonic stem (ES) cells transcriptional factors Oct4 and Nanog as well as surface markers of mesenchymal stem cells (MSC) such as CD105, CD73, and CD13. Nevertheless, they lack the expression of CD45, CD34, CD14, CD43, and of HLA-DR. These cells are able to undergo spontaneous and induced *in vitro *differentiation into osteoblasts, adipocytes and chondroblasts, muscle cells, and into neurons *in vitro*. After transplantation into normal mice, these cells show significant engraftment in liver, spleen, brain and kidney, among others [13].

Recently, hIDPSC, which are constituted by a homogeneous population positive for MSC markers, were shown to contribute for the reconstruction of large cranial defects produced in non immunosuppressed rats after their transplantation onto collagen membrane, without presenting any graft rejection [14]. Furthermore, populations of dental pulp MSC (DP-MSC) similar to those of hIDPSC, with immunosuppressive activity were described [15]. Analysis of their proliferation activity demonstrated that it was significantly higher in DP-MSC, when compared to those from bone marrow. Similarly to bone marrow MSC, these cells inhibited the proliferation of phytohemagglutinin stimulated T-cells, presenting an even stronger effect than in BM-MSC [15,16]. Other authors reported that DPSC can survive and engraft in ischemic environments in non immunosuppressed rats with acute myocardial infarction [17]. Taken together these data led us to investigate the myogenic potential of these cells in GRMD dogs.

Herein, we show results obtained after early transplantation of hIDPSC in four affected litter-mate GRMD dogs at an early age (two males and two females) with no use of immune suppression based on recent investigation. With this study we aimed at analyzing the cells ability for migration, engraftment, myogenic potential and expression of human dystrophin in affected muscles. Additionally, the efficiency of single and serial early transplantation were compared with the subsequent evaluation of the dog's clinical condition without the interference of immunosuppression protocols.

## Materials and Methods

### Dogs genotyping

Clinical studies were approved by the ethical committee of the School of Veterinary Medicine and Animal Science of São Paulo University. The animals were identified by numbers and experimental procedures were described in Table 1. DMD genotyping was done from blood genomic DNA extracted with GFX Genomic Blood DNA Purification Kit (GE Healthcare, Piscataway, NJ – USA/Canada) and established as previously reported [18]. DMD diagnosis was confirmed by restriction digestion of PCR products of the dystrophin gene with Sau96I and by elevated serum creatine kinase (CK).

**Table 1:** GRMD dogs, born April, 2006 used for IDPSC transplantation and respective controls.

Dog n°	Dog name	Cell Treatment	Onset treatment	Biopsy after 1^a ^injection	Progression/motility	Physical score		Outcome of experiment at time ...
						180d	365d	
♀	Laka 2L5	Control	-	0 d	Severe/major decline	4	†	Died (300d)
FC				107 d				Ascite
♀	Lancy 2L6	hlDPSC – S	Just with	107 d	Severe/major decline	3	†	Died (240d)
FT1-S			28 d					Ascite
♀	Amandita 2L4	hlDPSC – IM	Just with	107 d	Mild/modest decline	4	†	Died (480d)
FT2-IM			28 d					Cardiac failure
♂	Bis 2L2	Control	-	117 d	Severe/major decline	2	17	Alive
MC								
♂	Chokito 2L3	hlDPSC – S	Start with 44 d	47 d	Mild/modest decline	5	11	Alive and well
MT1-S								
			Followed by 8 injections/month	117 d				
				365 d				
♂	Toddy 2L7	hlDPSC – IM	Start with 44 d	47 d	Severe/major decline	6	† (24)	Died (370d)
MT2-IM			Followed by 5 injections/month	117 d				Gastric Malabsorption deficiency

All animals were descendant of the GRMD couple Peter (affected) × Lady (carrier)

### Mouse model

Normal mice were used to investigate the ability of hIDPSC migration by intraperitoneal injections and engraftment capacity of these cells without immunosuppression.

### Cell culture

Cells were obtained and characterized as described previously [13]. Shortly, dental pulp was extracted from normal exfoliated human deciduous teeth of 5- to 7-year-old children under local anesthetic. Tissue explant of dental pulp was used to isolate the cells. Human IDPSC cultures were maintained in DMEM/F12 (Dulbecco's-modified Eagle's medium/Ham's F12, 1:1, Invitrogen, Carlsbad, CA) supplemented with 15% fetal bovine serum (FBS, Hyclone, Washington), 100 units/ml penicillin, 100 units/ml streptomycin, 2 mM L-glutamine, and 2 mM nonessential amino acids. Cells were maintained semi-confluent to prevent their differentiation and replaced every 4 or 5 days. Medium was replaced daily. Human IDPSC were incubated at 37°C in a high humidity environment with 5% CO2.

### FACS analysis

Monoclonal anti-human SH2 (CD105, Serotec, Oxford, UK), SH3 and SH4 (CD73) (Case Western Reserve University, Cleveland, Ohio, USA) antibodies against cell surface molecules and their respective isotype controls were used in flow cytometry analysis. About 10^6 ^cells were incubated with primary antibody for 30 minutes at 4°C and washed in PBS with 2% FBS and 1 M sodium azide (buffer) followed by addition of secondary anti-mouse-PE – conjugated antibody according to manufacturer's instructions (Becton Dickinson, NJ, USA; Guava Technologies, CA). Negative controls were also performed incubating the cells in PBS followed by incubation with respective secondary antibody only. Results were analyzed using Guava Express^®^Plus software (Guava Technologies, Hayward, CA, USA). Flow cytometry was analyzed using a fluorescence-activated cell sorter (FACS, Becton, Dickinson, San Jose, CA) with CELL Quest program (Becton, Dickinson, San Jose, CA).

### In vitro myogenic differentiation of hIDPSC

Cells were cultured in DMEM-HG medium supplemented with 10% fetal bovine serum (FBS, Hyclone, Washington), 5% horse serum, 0.1 M dexamethasone, 50 nM hydrocortisone (Sigma Aldrich, São Paulo, SP), 1% antibiotic (100 units/ml penicillin and 100 mg/ml streptomycin; Invitrogen, São Paulo, SP) for 60 days.

### Fluorescent dye staining of hIDPSC

The culture cell was washed twice in calcium and magnesium-free Dulbecco's phosphate-buffered solution (DPBS, Invitrogen) and dissociated with 0.25% trypsin/EDTA solution (Invitrogen). The suspension was centrifuged and the cell pellet was ressuspended in DMEM (Invitrogen) with 10% FBS (Invitrogen) containing the fluorescent dye (Vybrant CM-Dil Cell-Labelling Solution; Molecular Probes, Invitrogen). Cells were incubated for 15 minutes at 37°C, washed twice in DPBS immediately prior to intraperitoneal injection in mice.

### Muscle biopsy and histology

Biceps femoralis biopsy samples were taken before the hIDPSC transplantation experiment on day 0 (d 0) and after 47, 107 and 117 days in different animals as summarized in Table 1. A second biopsy was taken from the male under systemic SC, delivery (MT1-S), after one year of treatment. Each biopsy from *biceps femuralis *was divided into 3 fragments for histological, immunofluorescence/WB and FISH analyses. For IF analysis, tissue samples were embedded in Jung Tissue Freezing Medium (Leica Mycrosystems Nussloch GmbH, Nussilosh, Germany) and frozen in liquid nitrogen. For WB analysis, 2 mm^2 ^fragments were frozen directly in liquid nitrogen in small flasks. For histological analysis routine hematoxylin and eosin (HE) staining was applied.

### Immunohistochemistry and Confocal microscopy

Differentiated cells were fixed in 4% paraformaldehyde for 2 h at 4°C, washed three times in wash buffer (150 mM NaCl, 1 mg/ml BSA, 0.5% Nonidet P- 40, 50 mM Tris pH 6.8) and membranes were permeabilized with two 10 min incubations in RIPA (150 mM NaCl, 1% Nonidet P-40, 0.5% sodium deoxycolate, 0.1% SDS, 1 mM EDTA, 50 mM Tris pH 8.0). Cells were subsequently re-fixed in 4% PFA for 30 min at 4°C, blocked in wash buffer for 1 h at 4°C and incubated overnight at 4°C with primary antibodies. Cy3-conjugated goat anti-(mouse IgG) secondary antibody and FITC-conjugated goat anti-(rabbit IgG) secondary antibody, normal mouse IgG and normal rabbit IgG were used. The primary antibody was omitted from some slides to serve as a negative control.

Myogenic differentiation was characterized using mouse anti-human muscle α-actinin antibody and rabbit anti-human muscle myosin antibody (Santa Cruz Biotechnology, CA, USA) and by RT-PCR using MyoD1(MD1) forward primer 5'AAGCGCCATCTCTTGAGGTA3' and reverse primer 3'GCCTTTATTTTGATCACC5' following the protocol described previously[13].

Tissues samples were excised, immediately frozen in liquid nitrogen and then stored at -80°C. Cryostat tissue sections with 5 μm thickness were mounted on a glass slide. According to the antibody used, slides were fixed and dehydrated with cold methanol (Merck, Darmstadt, Germany). To detect the presence of hIDPSC mouse, anti-hIDPSC antibody previously obtained [13] was used at a 1:500 dilution, whereas mouse anti-human nuclei antibody (Monoclonal, Chemicon International, California, USA) was used at a 1:100 dilution followed by FITC-conjugated secondary antibodies of respective isotype at a 1:1000. Dystrophin analysis was done using IF by applying the methodologies from our group [19]. Human specific anti-dystrophin monoclonal Mandys106 2C6 antibody was kindly provided by Dr. Glenn E. Morris at Center for Inherited Neuromuscular Diseases, Oswestry, UK [20]. Antibody DYS2 (Vector Laboratories – Burlingame, CA) against the C-terminal domain of dystrophin and anti-mouse IgG Cy3- conjugated secondary antibody were also used to confirm the presence of some positive dystrophin fibers. Microscope slides were mounted in Vectashield mounting medium (Vector Laboratories) with or without 4',6-Diamidino-2-phenylindol (DAPI). IF analysis was done in a Zeiss Imager Z1 Apotome microscope with epi-fluorescence, or using an argon ion laser scan microscope LSM 410 (Zeiss – Jena, Germany).

Confocal microscopy: An argon ion laser set at 488 nm for FITC and at 536 for rodamine excitation were used. The emitted light was filtered with a 505 nm (FITC) and 617 nm (rodamine) long pass filter in a laser scan microscope (LSM 410, Zeiss – Jena, Germany). Sections (5 mm) were taken approximately at the mid – height level of tissues. Photo-multiplier gain and laser power were kept constant throughout each experiment.

### Fluorescent in situ hybridization (FISH)

FISH analysis was done with specific centromere probes for human chromosomes X (green) and Y (red) (Aquiarius-Cytocell, Cambridge, UK). Tissue samples were fixed in 4% paraformaldehyde, embedded in Jung Tissue Freezing Medium and 5 μm sections were made.

The slides with sections were then fixed with cold methanol (Merck, Darmstadt, Germany) and dehydrated in series of ethanol. FISH reactions were done according to the manufacturer's protocol. Microscope slides were mounted in antifade (Vectashield mounting medium) with or without Propidium Iodide (PI, Vectashield mounting medium/PI). FISH analysis was made using confocal microscope as described above.

### Biochemical analysis

Dogs underwent periodic veterinary examinations during treatment. Blood samples were collected monthly by jugular venipuncture. Hematological and serum biochemical testing were performed with an automated cell counter (Baker System 9000, Serno-Baker Diagnostics Inc, Allentown, Pennsylvania). Urea, alanine aminotransferase (ALT), alkaline phosphatase (ALP), creatine kinase (CK) and creatinine were measured in serum with an automated analyzer (Labtest^® ^– LABTEST Diagnóstica S.A. – Lagoa Santa, MG). Data are shown in Table 2.

**Table 2:** Serum biochemical analyses in four GRMD dogs under hIDPSC therapy (MT1, MT2, FT1 and FT2) and matched age control (MC and FC).

**ANIMALS 30 days**	**CK (U/L)**	**ALT (U/L)**	**FA (U/L)**	**Urea (mg/dL)**	**Creat (mg/dL)**	**RBC (mlh/mm^3^)**	**Ht (%)**	**Hb (g/dL)**	**WBC (mil/mm^3^)**
Reference values*	*0–200*	*until 50*	*until 130*	*until 40*	*until 1.5 – 2*	*3.5 – 6*	*26 – 39*	*8.5 – 13*	*8,5 – 17,3*
FC	9,275	162	500	31	1	3.3	27	7.1	19,300
FT1-S	9,274	159	478	28.6	1	3.6	25	7.6	26,300
FT2-IM	4,647	134	352	22	0.98	4	26	8.8	32,000
MC	3,618	110	260	32.8	1	4.2	30	8.8	32,300
MT1-S	7,716	230	174	30	1	4.6	34	11	24,800
MT2- IM	5,668	163	217	32.9	1.1	4.7	33	11.5	23,600
**ANIMALS 75 days**	**CK (U/L)**	**ALT (U/L)**	**FA (U/L)**	**Urea (mg/dL)**	**Creat (mg/dL)**	**RBC (mlh/mm^3^)**	**Ht (%)**	**Hb (g/dL)**	**WBC (mil/mm^3^)**
FC	32,599	294	300	46	0.85	4.8	29	9.2	28,200
FT1-S	15,100	245	161	43.5	0.92	4.3	26	8.7	34,800
FT2-IM	24,784	192	117	37.8	0.9	4.6	28	9.2	30,200
MC	15,257	189	138	37.8	0.9	4.1	27	8.9	36,600
MT1-S	30,729	293	181	42.8	0.94	4.6	29	9.9	25,900
MT2- IM	15,291	237	191	46.9	0.92	4.6	28	9.7	39,100
**ANIMALS 180 days**	**CK (U/L)**	**ALT (U/L)**	**FA (U/L)**	**Urea (mg/dL)**	**Creat (mg/dL)**	**RBC (mlh/mm^3^)**	**Ht (%)**	**Hb (g/dL)**	**WBC (mil/mm^3^)**
Reference values*	*0–200*	*until 50*	*until 130*	*until 40*	*until 1.5 – 2*	*5.5 – 7*	*34 – 40*	*11 – 15.5*	*8 – 16,000*
FC	56,736	416	58	40	0.4	5.5	39	12.4	16,200
FT1-S	48,960	396	28	23	0.5	5.4	38	12.1	15,500
FT2-IM	51,296	228	8	38	0.4	6.0	39	13.1	14,100
MC	56,736	524	91	51	0.5	5.6	37	12	20,400
MT1-S	25,632	440	66	55	0.4	5.5	36	12.5	18,600
MT2- IM	36,512	500	62	40	0.4	5.5	38	12	15,600
**ANIMALS 360 days**	**CK (U/L)**	**ALT (U/L)**	**FA (U/L)**	**Urea (mg/dL)**	**Creat (mg/dL)**	**RBC (mlh/mm^3^)**	**Ht (%)**	**Hb (g/dL)**	**WBC (mil/mm^3^)**
reference values*	0–200	until 50	until 130	until 40	until 1.5 – 2	5 – 8	37 – 54	12 – 18	6 – 15,000
FC**									
FT1-S **									
FT2-IM	35,712	764	8	35	0.4	6.1	41	13.1	14,000
MC	41,920	376	49	48	0.6	5.6	36	13	20,000
MT1-S	40,384	604	41	34	0.5	5.2	33	12	18,000
MT2- IM***	9,280	208	33	50	0.7	5.1	34	11.7	15,000
**GRMD Control Data (n:6)**	35,710	551	43.25	35.75	0.52	5.75	37.75	12.87	14,6
**Normal Dogs Data (n: 6)**	145	15	82	28	1.8	4.2	30.7	14.2	11,7

Also, control dogs were used from all colony animals (Normal X Affected)

*Reference values from Veterinary Hospital (HOVET) – FMVZ-USP and FMVZ-UNESP, São Paulo, Brazil.

** Animal died with 10 months (FC) and 8 months (FT1-S) *** Animal died with 13 months

### Physical score exams

We established a physical exam score (Table 3), adapted from literature [21] that evaluated dogs motility and posture, and which gave values between 0 (normal) to 24 (severely affected).

**Table 3:** Physical exams score for motility and postural features from dystrophic dogs

**Scores**			
**CRITERIA**	**0**	**1**	**2**

**1. Posture symmetry**	Symmetric animal	Slight asymmetry: discreet lordosis	High asymmetry: lordosis posture and tail elevation
**2. Increase of tarsal normal angle (plantigrady)**			
	Absent	modest	severe (walk on tarsus)
**3. Increase of carpal normal angle (palmigrady)**			
	Absent	modest	severe (walk on carpus)
**4. Stifle stiffness while moving**	normal stifle motility	slight stifle stiffness	severe stifle stiffness associated with short strides
**5. Pelvic balance**			
	Normal	reduced	absent
**6. Postural tone (muscle palpation and inspection)**			
	normal tone	hypotonic	flaccid
**7. Contractures**	Absent	decrease in passive range of motion	contractures limiting passive range of motion
**8. Hopping**			
	Normal	reduced	inability to achieve postural reaction
**9. Standing up**	Normal	ability reduced (perform task slowly/slower)	
	inability to stand up alone		
**10. Barrier crossing**	normal	reduced	inability to jump
**11. Food prehension**	Eat normal dry food	changed for wet food	laborious swallowing
**12. Locomotion apparatus structure**	strong	losing structures components as muscle mass and bone fragilities	weak and thin

Total score = 24			

## Results

### Evaluation of hIDPSC skeletal myogenic potential and migrating ability

**Figure 1:**
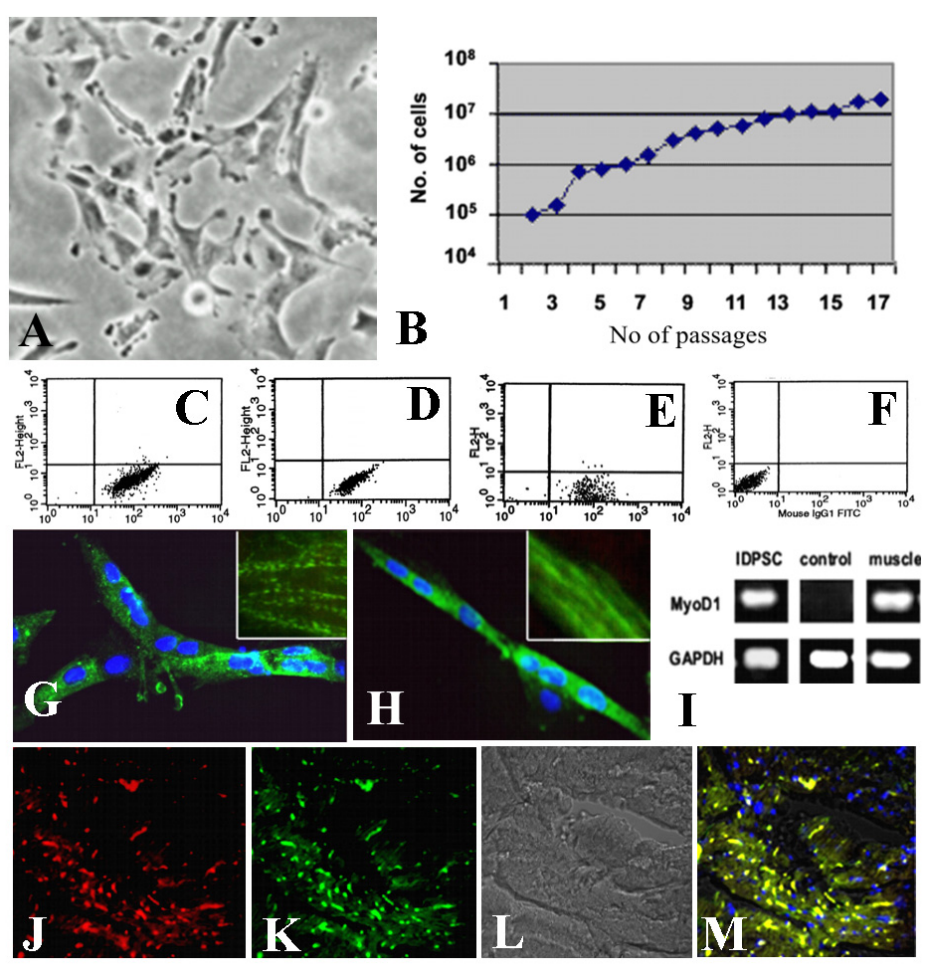
**Characterization, myogenic differentiation *in vitro *and migrating capacity in vivo of hIDPSC.** (A) Fibroblast-like morphology of hIDPSC. (B) Proliferating potential of these cells during 16 successive passages. (C-E) Flow cytometry showing expression of CD105 (SH2), CD73 (SH3 and SH4), respectively. (F) Negative control for respective isotype. (G,H) Myogenic differentiation *in vitro*: fused hIDPSC forming myotubes. Positive immunostaining with α-actinin (G) and myosin (H): insets in (G and H) show details of anti-bodies localization within myotubes, higher magnification. (I) RT-PCR analysis of the expression of human dystrophin (MyoD1) observed hIDPSC and human muscles, used as a positive control. (J-M). Migrating capacity of hIDPSC visualized 30 days after their intraperitoneal injection into normal mice (J-M): (J) Cells stained with DiI-Vybrant (red) in mouse, (K) positive reaction with primary human anti-IDPSC antibody in mice (secondary antibody FITC-conjugated was used (green), (L) Morphology of mouse cardiac muscles. (M) merged image of J-L. A= light microscopy, phase contrast, G-H = epi-fluorescence (EF), J-M= confocal microscopy: J, K = fluorescent microscopy (Fcm), L = DIC (Differential interference contrast) M = Fcm+DIC. Scale bars: G,H = 10 μm, A,J-M = 100 μm, N-P = 50 μm

Isolated hIDPSC (2n = 46, XY) were used at passages 6 or 7 [13]. Because cells have been cryopreserved, we checked their morphology (Figure 1A), proliferation efficiency (Figure 1B) and expression of principal MSC markers such as SH2, SH3 and SH4 (Figure 1C–F), prior to their use. Additionally, cells were re-analyzed prior to transplantation. Their *in vitro *ability to differentiate into myotubes was confirmed by immunostaining of α-actinin (sarcomeric) (Figure 1G) and of myosin (Figure 1H) and by RT-PCR of the MyoD1 gene (Figure 1I). After thawing, migration ability of CM-DiI-fluorescently labeled hIDPSC (10^5^) was confirmed by its detection in the heart muscle of intraperitoneal injected normal mice after 2 weeks (Figure 1J–M). No prior immunosuppression was done.

### hIDPSC transplantation in GRMD dogs

**Figure 2:**
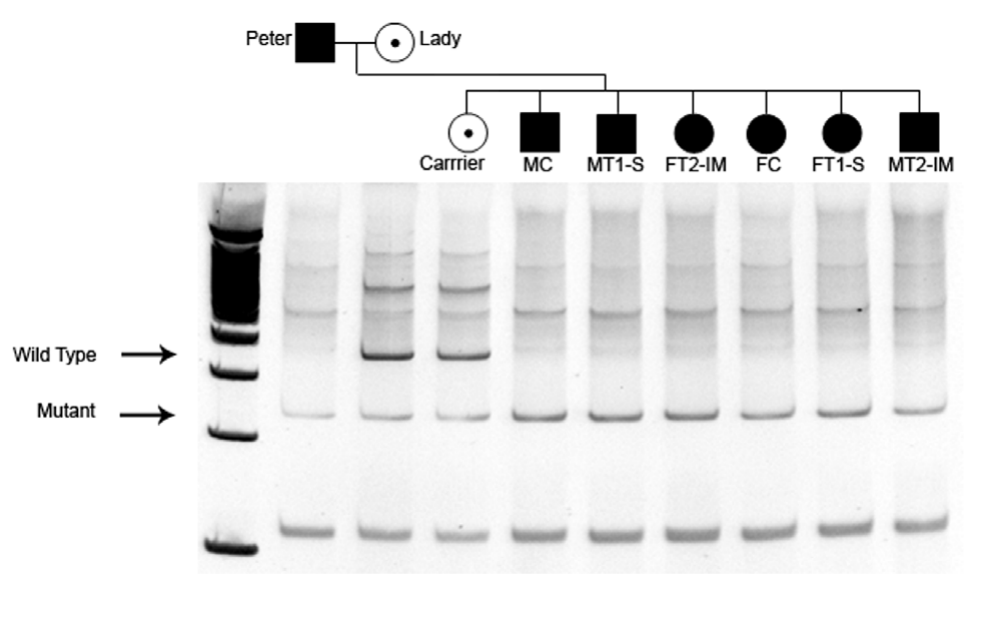
**DMD genotyping.** GRMD puppies from a colony of dogs with X-linked muscular dystrophy were genotyped within 48 hs after birth. The genomic PCR product digested with Sau96I produces the wild type band (310 pb) and the mutant band (150 pb) labelled with arrows. ■ = Affected male. ● = Affected female. ◉ = Carrier female.

To minimize the effects of inter and interfamilial variability previously observed in GRMD dogs we used animals from the same litter. Six affected animals (3 males and 3 females) born after the breading of an affected male with a carrier female through artificial insemination were used in the experiment. The dystrophin mutation in these puppies was confirmed by PCR (Figure 2). Cells were injected in four dogs, two MT1-S (male transplanted 1- systemic) and MT2-IM (male transplanted 2- intramuscular) and two females FT1-S (female transplanted 1-systemic) and FT2-IM (female transplanted 2- intramuscular). Each gender received 6 × 10^7 ^hIDPSC via arterial or muscular injection. One non-injected male (MC – male control) and one non-injected female (FC – female control) were analyzed as age-matched controls (Table 1). The first systemic and local injections were done respectively in two 28- days-old female dogs. To verify the potential effect of short- and long-term treatment with hIDPSC, females received one unique injection, whereas males were treated with monthly injections, starting with 44 days old.

One male (MT2-IM) received six intramuscular (*biceps femularis*) injections, whereas another subject (MT1-S) received nine arterial (femoral artery) systemic injections. No immunosuppression was used before or after cell transplantation. Muscle biopsies were obtained at the beginning of the experiment (d 0) from FC only; after 107 days in the three females and after 47 and 117 days only from the injected male dogs. After one year, a muscle biopsy was collected from MT1-S.

### Engraftment of hIDPSC in muscles of GRMD dogs after transplantation

**Figure 3:**
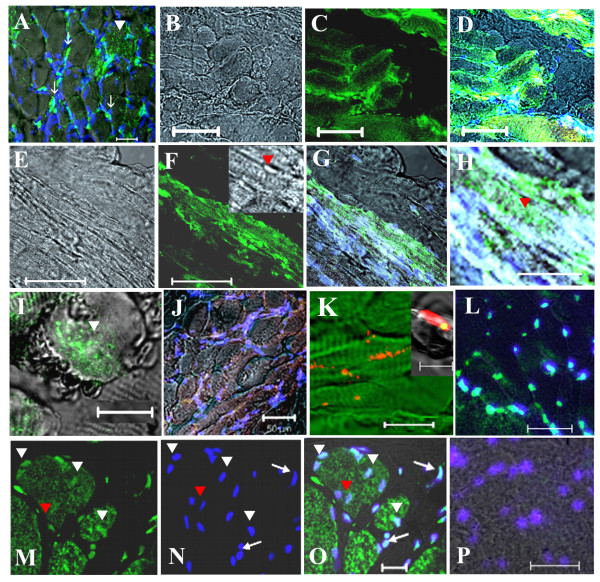
**Representative figures of hIDPSC engraftment observed within canine skeletal muscles (GRMD).** (A) FT2-IM, after 107 days of single transplantation: Positive immunostaining with anti-hIDPSC antibody (green) was observed in several muscle fibers (white arrowhead) and in the nuclei (white arrows, blue, DAPI stained superposition with anti-IDPSC antibody, green) of hIDPSC localized in the periphery of canine muscle fibers. (B-I) One year after multiple hIDPSC transplantation. Positive immunostaining with anti-IDPSC antibody was observed in MT1-S muscles: (B-D) transversal and (E-H) longitudinal sections. Inset in (F) demonstrate (higher magnification) skeletal muscle Z-bands (red arrowhead) observed in the local of positive immunostaining with anti-hIDPSC antibody (DIC). (H) Higher magnification of (G) demonstrating positive reaction of hIDPSC antibody (green) with skeletal muscle Z-bands (red arrowhead). (I) Chimeric human/canine muscle fiber only a half of which presents positive green fluorescent immunostaining (green). (J) Control: affected male without hIDPSC transplantation immunostained with anti-hIDPSC antibody did not present any labeling. (K) FISH analysis of dystrophic male's muscles using specific human probe for chromosome: Y (red) and in inset X (yellow, as a result of merged images of PI (red) stained nucleus and probe of chromosome X (green) are presented). (L-P) Immunostaining using FITC conjugated anti-human nucleus (anti-HN) antibody (green). (L) Positive control. Merged image of positive staining with anti-HN antibody and nuclei stained with DAPI in normal human muscles. (M-O) Positive immunostaining with anti-HN antibody observed in the nuclei of hIDPSC (green) engrafted into canine muscle fibers of MT1-S. They (white arrowhead) can be seen within canine muscle fibers and in perimysium (white arrows). Canine nuclei (group of 4 nuclei indicated by red arrow) did not present any reaction with anti-HN antibody. (P) Negative control. Muscles of normal dog did not react with anti-HN antibody. Only nuclei stained with DAPI can be observed. Confocal microscopy, A,D,G-L,P = Fcm+DIC; C,F,M,N = Fcm, B,E, Inset in (F) = DIC, Scale bars: A-H, L,P = 50 μm, K = 100 μm, and M-O = 20 μm, Inset in (K) = 10 μm

Serial frozen sections of canine muscles were analyzed for engraftment. Scattered human cells were identified in females who received one injection of hIDPSC each. Only some of the sections obtained from these animals showed a few human anti-hIDPSC antibody positive fibers along with the presence of human cells in muscles and connective tissues, and probably in the region of injection from dog FT2-IM, representative by Figure 3A. MT1-S presented an apparently higher engraftment of hIDPSC in muscle fibers, as demonstrated by anti-hIDPSC antibody, which were detected in several sections of the biopsies (Figure 3B–J). Transversal sections (serial-section analysis) from canine/human chimeric muscle fibers showed variable engraftment within different fibers as well as within one fiber, which indicates the occurrence of fusion between both cell types (Figure 3B–D,I,J). FISH analysis with human Y and X DNA probes confirmed these results and showed their presence in chimeric muscle fibers. Interestingly, FISH experiments showed human nuclei in the central part of the fibers; in a pattern resembling those of immature myotubes (Figure 3K and inset). Further analysis using FITC-conjugated anti-human nuclei specific antibody also showed the presence of human nuclei in several MT1-S muscle fibers (Figure 3L–P).

### Human dystrophin expression in muscles of GRDM dogs

**Figure 4:**
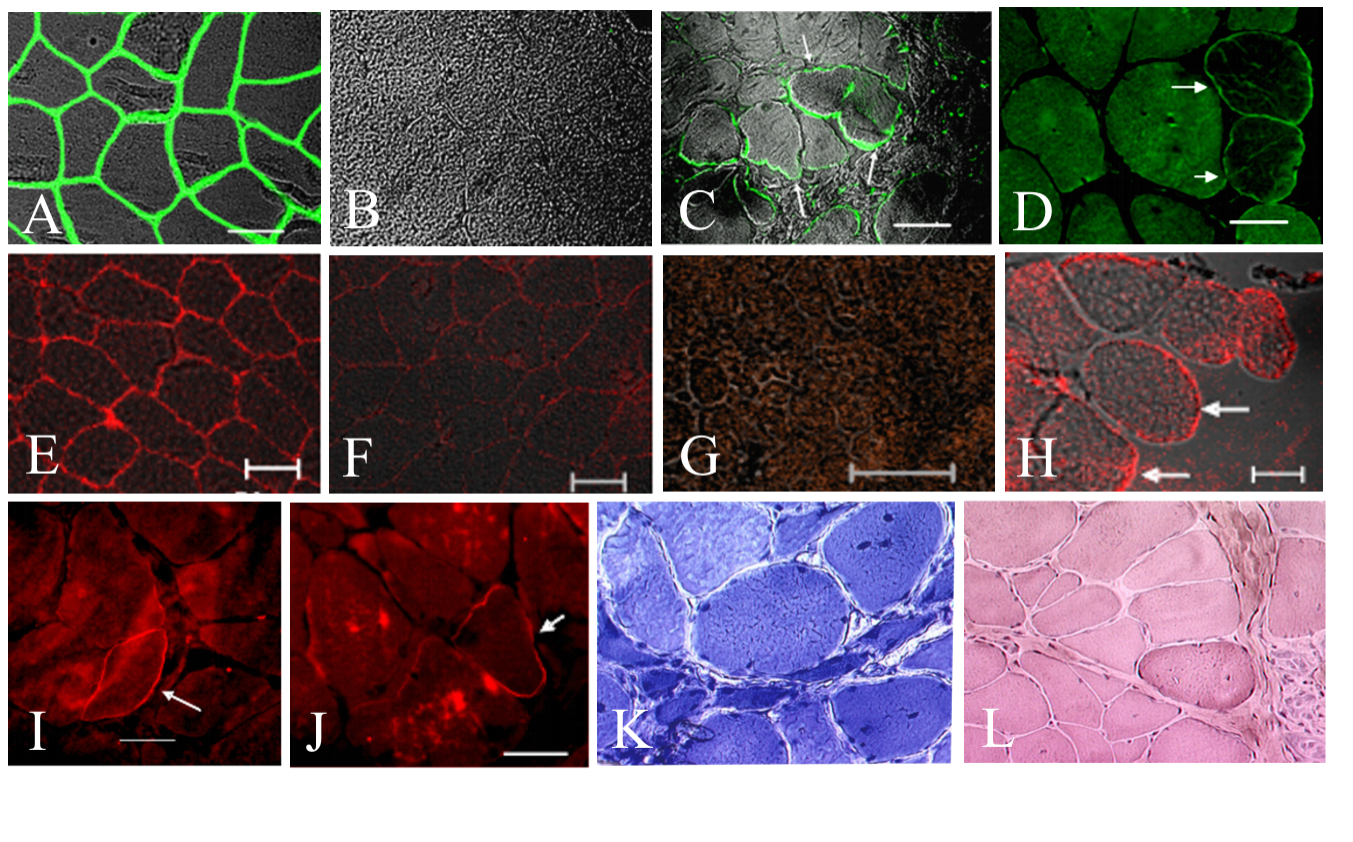
**Immunofluorescence analysis using the specific human anti-dystrophin monoclonal antibodies: Mandys106 2C6 (A-D) and C-terminal (E-J) antibodies one year after the hIDPSC transplantation.** (A) Positive control: expression of Mandys106 2C6 antibody in normal human muscles. (B) Negative control: lack of expression of same antibody in the muscles of normal dog. (C, D) MT1-S shows positive staining with Mandys106 2C6 in large dystrophic fibers (white arrows). (E) Positive control: expression of C-terminal in normal human muscles. (F) C-terminal antibody in the muscles of normal dog presents weak labeling. (G) Negative control: lack of its expression in the muscles of affected dog. (H-J) MT1-S shows positive staining with C-terminal antibody in some large dystrophic fibers (arrow). K) Toluidine blue and L) HE staining shows very large fibers with multiple centrally located nuclei and splitting. A, C, E-H = Fcm + DIC, B = DIC, D,I,= EF, J,K = Light microscopy, Scale bars = 50 μm

After 107 and 117 days, immunofluorescence (IF) analysis of muscle biopsies from the four injected dogs detected few fibers labeled with human specific anti-dystrophin antibody (Mandys1062C6), (data not shown). However, scattered large fibers positive for this antibody were observed after one year of consecutive injections in MT1-S (Figure 4A–D). The expression of dystrophin in this animal was also confirmed with a C-terminal antibody for dystrophin (Figure 4E–J). However, the expected band of 427 kDa was not observed in western blot (WB), which may indicate that only a small amount of the expressed protein was present in the sample (data not shown).

### Histological characterization of GRMD dogs after treatment with hIDPSC

Muscle degeneration showing necrosis, splitting, centrally located nuclei, and significant endomysial and perimysial connective tissue replacement was observed in all injected and not injected muscles. Interestingly, muscle histopathological analysis of MT1-S done one year after the first injection showed a high number of very large fibers, with several centrally located nuclei associated to a significant proportion of splitting fibers which is suggestive of segmental necrosis (Figure 4K,L). FISH and anti-human nuclei specific antibodies results supported that hIDPSC can contribute for the formation of these large centrally nucleated fibers (Figure 3K,M–O).

### Clinical and laboratorial assessment

Several biochemical parameters were assessed in all dogs monthly and analyzed at times 30, 75, 180, 360 days (Table 2). Mean serum CK showed a peak between 75 to 180 days with an apparent stabilization afterwards. Other biochemical parameters summarized in Table 2 suggest that all treated dogs maintained liver and kidney functions and apparently did not show any evident alteration in response to cells transplantation such as white blood cell counting (WBC).

The progression of the dystrophic process was analyzed based on a mobility score which is summarized in Table 1. One affected female control (FC) and two dogs under treatment died during the experiment (FT1-S and MT2-IM). The motility score showed a moderate disease progression in dogs MT1-S and FT2-IM. Although when comparing male and female phenotypes, it should be taken into consideration that females locomotion apparatus differs from males since they have a lighter bone structure, a smaller body and a more delicate locomotion pattern. MT1-S, at one year old, showed a good performance with moderate scores mainly in postural tone, standing up, crossing barriers and hoping. Currently, at age of 18 (Additional file 1) and 25 months (Additional files 2 and 3) his motility is stable and he is doing well. In our experience almost 90% of the GRMD dogs die before 24 months of age. However, Chokito, the dog in which we found a greater amount of muscle dystrophin is showing a better course. The movies show this dog at 18 and 26 months old and so far he showed no decline.

FT2-IM had a comparable performance but she died suddenly of cardiac arrest at age of 16 months (Table 1, 2, 3).

## Discussion

Herein we showed that hIDPSC were capable to migrate and engraft into GRDM dog muscle as confirmed by FISH and immunohystochemical analyses. Due to our ethical committee rules which is against dogs euthanasia or taking multiple biopsies we did not quantify the engraftment of these cells into different muscles, however all analyzed biopsy fragments demonstrated the presence of hIDPSC. We observed that these cells were able to form chimeric canine/human fibers with recipient muscles, although the expression of human dystrophin was observed only in limited number of the fibers following early, multiple cell transplantation. Additionally, these cells persisted in the host muscle for at least 1 year. Moreover, it has been suggested that thawing might modify the properties of SC [22].

Two independent groups have previously reported the transplantation of different types of canine stem cells in the GRMD model. In the first study, 7 dogs received canine allogenic hematopoietic cell transplantation at ages 4.5 to 5.5 months [10]. However, donor cells did not show any significant contribution to the injured skeletal muscle nor significant increase of dystrophin positive fibers, although near-complete or complete donor hematopoietic chimerism was detected in physically active dogs. The authors concluded that allogenic bone marrow cell transplantation is not a viable therapy in its current form due to the absence of clear clinical benefits [10]. The second group used canine heterologous wild type (WT) mesoangioblasts and allogenic genetically modified mesoangioblasts [11]. The authors reported GRMD muscle function improvement and high level of dystrophin expression in particular after heterologous wild-type (WT) mesoangioblasts transfer, but less with autologous genetically modified SC. Both early and late SC transplantation showed very promising results [11]. However, since immunosuppression was applied in both studies; clinical interpretation of the results is difficult because the use of anti-inflammatory molecules may improve muscle function in affected muscular dystrophy patients [12].

The number of animals, which received SC transplantation in present work, was just the same used in previous publications [10,11]. However, our study differs in several aspects. First, we used in our experiments an exceptional litter with 3 affected females and 3 affected males. Second we transplanted human, not canine SC without the interference of immunosuppression protocols. Third, clinical monitoring of dogs showed no signs of fever, skin eruption, arthralgia or even glottis edema suggesting, that human cells were well accepted by the dog organism.

As canine mesoangioblasts and hIDPSC are derived from different tissues and species, it is difficult to compare their potential benefits after transplantation in the GRMD model. However, both cell types proliferate efficiently and present a good migrating capacity in host tissues [10,11]. In our experiment, in contrast to the study reported by Cossu group [11] we did not observe significant increase of dystrophin positive fibers in the 4 GRDM dogs after SC transplantation. Although all dogs showed engraftment of hIDPSC in muscles, only one of them (MT1-S) showed human dystrophin expression. However, this dog, which received the largest amount of human stem cells, is still alive and well at 25 months of age after the experiment was over. It showed a slower rate of progression of the dystrophic process than his five litter-mate dogs or to most of the dogs from the Brazilian GRMD colony, since the majority of them died before 18 months of age (unpublished observation).

At the end of the experiment, MT1-S had no difficulties in swallowing or chewing hard food, maintained jaw strength as well as the ability to run while carrying objects. The female FT2-IM had also good clinical scores despite the fact we could not detect dystrophin in her muscle biopsy. It died suddenly of cardiac failure at 16 months of age. Due to females having a more delicate structure of the locomotion apparatus and other anatomical variation in the thorax and muscle structures [23], gender could indeed have interfered with muscle functions and motility performance. Furthermore, since female GRMD dogs are very rare, there are no parameters to evaluate the natural history of their disease, not to mention the absence of studies that use such dogs. FC, FT1-S, FT2-IM and MT2-IM died from dystrophy complications at the ages 10, 8, 16 and 11 months, respectively.

In DMD patients, serum CK is elevated since birth and shows a peak of activity around 2 to 4 years when there is massive muscle degeneration [24]. In accordance with Sampaolesi studies [12] we observed that mean serum CK activities in GRMD dogs were higher between 75 to 180 days followed by an apparent stabilization (Table 2).

Significant immune modulatory function of MSC has been demonstrated in hematopoietic stem cell transplantation. They strongly inhibit alloantigen-induced dendritic cell differentiation, down-regulate alloantigen-induced lymphocyte expansion, decrease alloantigen-specific cytotoxic capacity mediated by either cytotoxic T lymphocytes or NK cells. Interestingly, a more effective suppressive activity on mixed lymphocyte culture-induced T-cell activation was observed when MSC were heterologous rather than autologous. It was suggested that due to these properties, MSC can be used to prevent immune complications related to both hematopoietic stem cells and solid organ transplantation and it was proposed that MSC are "universal" suppressors of immune reactivity. Moreover, it was shown that regulatory CD4+ or CD8+ lymphocytes were generated in co-cultures of peripheral blood mononuclear cells with MSC. This strongly indicates that these regulatory cells may amplify the reported MSC-mediated immunosuppressive effect [25-27]. In our experiments the dogs did not show any immune reaction to hIDPSC transplantation. This could be explained by the fact that according to previous publication immunosuppressive activity of human DPSC was significantly higher, when compared to those from bone marrow [15]. More recently, similar results were observed with different populations of stem cells isolated from dental pulp [14,15,17].

The absence of dogs immune response to hIDPSC transplantation here reported for the first time is an important observation, because anti-inflammatory drugs could occult the benefits of stem cells therapy for dystrophy [28]. Indeed, white blood cells counting did not present any important changes in response to cell transplantation and no lymphocytes infiltration was observed in muscle cells. We also did not observe immune reactions using the same cells in experiment on reconstruction of cranial defects in rats [14]. This result is also supported by other authors, which demonstrated that human stem cell isolated from dental pulp have immunosuppressive activity [15]. In conclusion, we believe that rejection of hIDPSC did not occur since they present all basic characteristic of mesenchymal stem cells [29].

It is very tempting to suggest that the presence of some dystrophin in muscle as a result of repeated systemic hIDPSC transplantation ameliorated the clinical condition of MT1-S since this disease in dogs causes their death usually between 11 and 18 months [9,30]. Interestingly, after transplantation of human DPSC in non immunosuppressed rats with acute myocardial infarction, surviving and engraftment of the cells in ischemic environments have been observed. Although no differentiation into cardiac or smooth muscle as well as into endothelial cells was observed, these cells apparently contributed for the improvement of left ventricular function, induced angiogenesis and reduced infarct size. According to the authors, the benefits observed after DPSC transplantation might be due to secretion of paracrine factors by these cells [17]. Since the expression of dystrophin was modest, clinical improvement in this dog might be the result of the immune modulatory effect of hIDPSC transplantation rather than the presence of dystrophin, or rather than the great clinical variability observed among GRMD dogs [31]. However, our preliminary results, which should be validated in a larger sample, suggested that multiple systemic transplantations of hIDPSC in GRMD dogs could slow down the progression of the dystrophic process, through similar mechanisms described by Gandia and co-workers [17].

In short, our investigation confirmed that early systemic delivery of SC in GRMD model is more effective than local injections. Although we showed that donor human SC can engraft, differentiate, and persist in the host, it seems that the apparent clinical benefit observed in treated animals is not due to dystrophin expression in the host muscles, but due to their immune modulatory effect of SC from dental pulp [14,15,17,32]. However, both observations that cell engraftment increases with consecutive transplantation and the absence of immunological response, have very important implications in designing future therapeutic trials. The potential clinical effect of SC in GRMD model should be supported by further investigation involving a larger number of animals and the efficacy of later as compared to early transplantation.

## Competing interests

The authors declare that they have no competing interests.

## Authors' contributions

All authors have read and approved the final manuscript. The specific contributions of each author are: DSM, TPG, ACM, MPB assisted clinical support to the dogs, conduce the clinical trial with laboratory analyses and physical scores establishment. EZ, NV conducted genotyping process, material collections and also immunohistochemistry and blotting dystrophy analyses. SASF, CMCM, OASA conduce the stem cells preparation and FISH analyses; AK conducted immunohistochemistry and confocal microscopy; RMC co-conducted the clinical trial and cell applications and assisted with dogs analyses; IK, CEA, MAM and MZ conceived, designed and directed the entire study, interpreted all data and wrote the manuscript.

## Supplementary Material

Additional file 1 MT1-S, at 18 months, showed a good performance with moderate scores. His capacity of running and strength with objects are well after cell therapy. Click here for file

Additional file 2 MT1-S, at 25 months, continues with a good performance. After 16 months of experimental end, Chokito continues with his capacity of running and muscle strength. Click here for file

Additional file 3 MT1-S with 25 months. Chokito presenting his capacity of jumping obstacles. Click here for file
